# *Parechovirus A* prevalence in adults in The Netherlands

**DOI:** 10.1007/s00705-020-04547-0

**Published:** 2020-02-14

**Authors:** Lieke Brouwer, Katja C. Wolthers, Dasja Pajkrt

**Affiliations:** 1 Laboratory of Clinical Virology, Department of Medical Microbiology, Amsterdam University Medical Centers, Location Academic Medical Center, University of Amsterdam, Meibergdreef 9, 1105 AZ Amsterdam, The Netherlands; 2 Department of Pediatric Infectious Diseases, Emma Children’s Hospital, Amsterdam University Medical Centers, Location Academic Medical Center, University of Amsterdam, Amsterdam, The Netherlands

## Abstract

Human parechoviruses (HPeV) of the species *Parechovirus A* are highly prevalent disease-causing pathogens in children worldwide. HPeVs are capable of causing severe disease in adults as well, but the prevalence in adults may be much lower. The aim of our present study was to determine the prevalence of HPeV in clinical samples from adults sent in for diagnostic procedures in a tertiary hospital in the Netherlands. From a total of 10,645 samples obtained from 6175 patients, 20 samples from 11 patients (0.18%) tested positive for HPeV by RT-PCR. Two patients were positive for HPeV-1, two for HPeV-3, and one for HPeV-6. Six HPeVs could not be typed. Eight of the 11 HPeV-positive patients were immunocompromised. Due to comorbidity, we were unable to attribute the patients’ clinical symptoms to the HPeV infection. The HPeV prevalence in adults found in this study is low compared to HPeV prevalence in children. This may be largely explained by the high seropositivity rates in adults, although there could be other mechanisms involved.

**Electronic supplementary material:**

The online version of this article (10.1007/s00705-020-04547-0) contains supplementary material, which is available to authorized users.

## Introduction

Human parechoviruses (HPeV) of the species *Parechovirus A* of the family *Picornaviridae* are known to circulate extensively in children worldwide [1]. HPeVs can cause mild respiratory or gastrointestinal symptoms, as well as severe neurological disease, such as meningitis and encephalitis. Overall, the clinical relevance of HPeV infections in adults remains largely unknown. Several studies have found a very low HPeV prevalence in adults compared to children [2–6], although cases of adults experiencing severe illness caused by HPeV have been described [7–10]. We performed a retrospective study to identify the HPeV prevalence in adults in a tertiary hospital in Amsterdam, The Netherlands, and to determine the clinical relevance of these viruses in adults.

## Materials and methods

Between January 1, 2008 and January 1, 2018, a total of 10,645 clinical samples from 6175 patients older than 18 years of age were tested for HPeV at the Amsterdam UMC, location AMC, a tertiary academic hospital in Amsterdam, The Netherlands. The samples included i.a. stool samples, nose/throat swabs, cerebrospinal fluid, and serum samples from patients with a variety of symptoms, including gastrointestinal, respiratory and neurological symptoms, and fever. The patients were either hospitalized or presented to the outpatient clinic of the AMC. The samples also included fecal samples from 93 healthy fecal donors. For all respiratory and stool samples, an in-house multiplex RT-PCR, including the HPeV 5′UTR, was performed routinely. Other samples were tested for HPeV when requested by the treating physician. For all positive samples, RNA was extracted [11], the viral protein 1 (VP1) region was amplified by a nested PCR, and a sequencing reaction was performed using a Big Dye Terminator Kit [12]. For the current study, the VP1 sequences were aligned with Mafft version 7 software [13], and a maximum-likelihood (ML) phylogenetic tree of the clinical strains and reference strains from the GenBank database was constructed using RAxML version 8.2.12 [14].

## Results

Of the 10,645 samples, 20 samples (0.19%), belonging to a total of ten patients and one healthy fecal donor without symptoms (11/6,175, 0.18%), tested positive for HPeV RNA. Information on gender, age and symptoms was recorded for all patients (Table 1). HPeV-positive patients reported respiratory, gastrointestinal and neurological symptoms, and fever. Out of the eleven cases, eight had an immunocompromised status due to (treatment for) hematologic malignancies.

**Table 1:** Characteristics and HPeV types of 11 patients with HPeV-positive samples

	N/total
*Gender*	
Male	6/11
Female	5/11
*Age*	
30–39	6/11
50–59	3/11
60–69	2/11
*Symptoms*	
Respiratory	4/11
Gastrointestinal	3/11
Neurological	1/11
Fever	4/11
Unknown	1/11
Healthy	1/11
*Immunocompromised*	
Yes	8/11
No	1/11
Unknown	2/11
*HPeV type*	
HPeV-1	2/11
HPeV-3	2/11
HPeV-6	1/11
Unknown	6/11
*Sample type*	
Stool	6/11
Throat swab	5/11
CSF	1/11
Serum	1/11

**Fig. 1:**
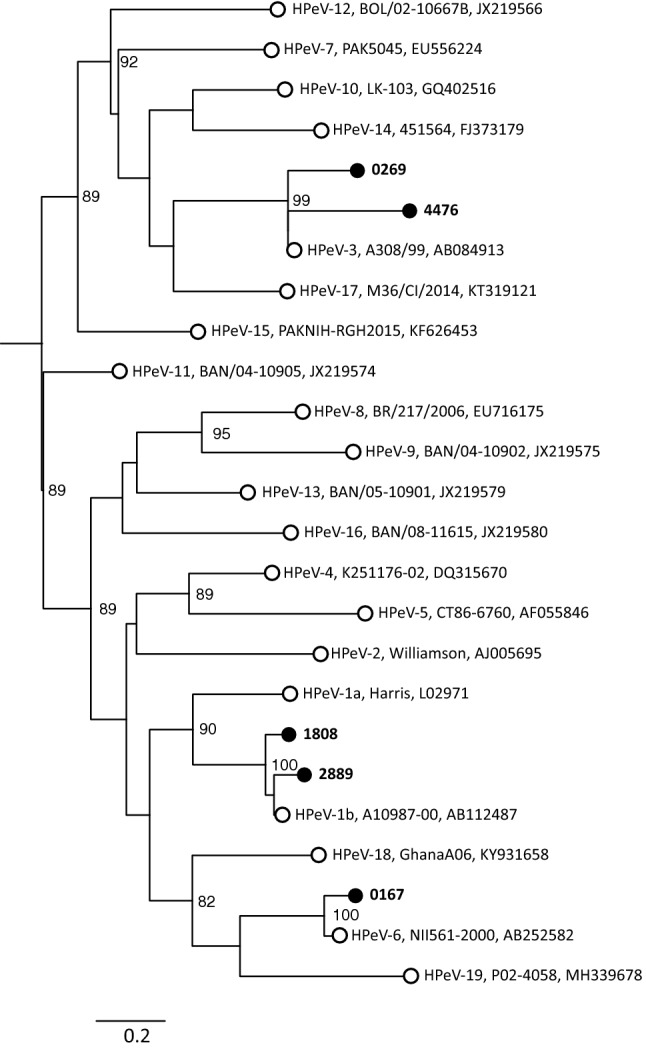
Phylogenetic tree including the study strains (closed circles) and reference strains (open circles) from the GenBank database. Phylogenetic analysis was performed on the VP1 sequence (nt positions 2336–3037 of the genome sequence of the Harris HPeV-1 strain, accession no. L02791) using the maximum-likelihood (ML) method with the generalized time-reversible (GTR) + gamma nucleotide substitution model (1000 bootstrap replicates). For the reference strains, the type, strain name and accession numbers are given. Bootstrap values ≥ 70% for the branches are shown

Of the eleven viral strains, two could be typed as HPeV-1, two as HPeV-3, and one as HPeV-6, while six could not be typed (Fig. 1). One patient had multiple positive stool samples over a period of over three months, one patient tested HPeV positive in both throat swab and stool, and one patient tested HPeV positive in both serum and stool. All three patients were immunocompromised.

The HPeV sequences obtained in this study were deposited in the DDBJ/EMBL/GenBank nucleotide sequence databases with accession numbers MK861915 through MK861917.

## Discussion

We found a remarkably low HPeV prevalence of 0.18% among adults in our hospital. This is comparable to the low HPeV detection rates previously reported for critically ill adult patients in the Netherlands (0.1%) [6] and adult cohorts in other countries (0–0.3%) [2–5]. The low prevalence is in contrast with the HPeV prevalence found in children, which is much higher [1]. Furthermore, the low HPeV prevalence is in contrast with the enterovirus (EV) prevalence in adults, which is also higher [15–18]. The latter might be the result of the vast number of EV serotypes (> 100), of which dozens are known to circulate on a large scale. It is possible that children do not encounter all circulating serotypes during childhood and remain susceptible to several EV types as an adult. For HPeVs, only types 1 and 3 are highly prevalent. Most children would gain immunity against both of those HPeV types at a very young age [19–21]. This humoral immunity is thought to be lifelong and would thus protect the vast majority of adults against HPeV infection.

There are additional mechanisms that might protect adults against infection with HPeV. Although cross-immunity is not presumed to occur in EV infection, it could play a role in the immune response against HPeVs, as supported by data from previous studies [22]. In a cohort of mothers with HPeV-infected children, we reported previously that an active infection was found in seven of 24 (29%) mothers–a much higher prevalence than in our current study, indicating that HPeV infections do occur in adults [21]. A shorter period of viral shedding may explain why these HPeV infections in adults are often missed.

Notably, eight out of the 11 HPeV-positive individuals in our study were known to be immunocompromised. In two of the immunocompromised patients, the infection was disseminated, with multiple sample types testing positive for HPeV. In one immunocompromised patient, the infection was prolonged, lasting for over three months. This suggests that immunocompromised individuals might be at a higher risk of HPeV infection, but also at a higher risk of experiencing an abnormal course of the infection. This could be a direct effect of compromised humoral immunity due to disease or treatment. Since most of our patients suffered from (severe) comorbidity, we cannot make any conclusions on the symptomatology of HPeV in these patients.

In conclusion, we found the prevalence of HPeV among adults in our hospital to be 0.18%. The majority of the HPeV-infected patients had a severe (humoral) immunodeficiency. This is in line with the current belief that protection against HPeV is mediated mainly through neutralizing antibodies, although other mechanisms may be involved in adults.

## Electronic supplementary material

Below is the link to the electronic supplementary material.

Supplementary file1 (SVG 32 kb)
